# Comparing Articaine brands: A randomized non-inferiority controlled trial

**DOI:** 10.1016/j.heliyon.2021.e07252

**Published:** 2021-06-09

**Authors:** D. Arboleda-Toro, L. Toro, Y.A. Osorio-Osorno, L. Castrillon-Pino, N.M.V. Florez-Zapata

**Affiliations:** a Facultad de Odontología, Universidad de Antioquia, Medellín, Colombia; b Universidad EIA, Envigado, Colombia; c Cancer Research Institute, Biomedical Research Center of the Slovak Academy of Sciences, Bratislava, Slovakia

**Keywords:** Local anesthetics, Amides, Pain, Oral surgery, Nerve block, Clinical studies

## Abstract

**Introduction:**

The substitution of reference drugs for similar, new or existing drugs in the market is a dilemma present in daily dental procedures. In order to decide whether or not to adopt a new drug in relation to the reference, a clinical trial can provide the necessary evidence.

**Methods:**

A total of 179 healthy volunteers (18–25 years) requiring extraction of mandibular third molars completed the study. Subjects were randomized into 4 groups: right, left, Arteek-SP and Septanest. Allocations consisted of 4% Articaine hydrochloride with 1:10000 epinephrine. The primary aim of this randomized controlled trial was to determine whether the test drug Arteek-SP was noninferior by a margin of 10% in the proportion of patients with total absence of pain, compared to the reference drug Septanest in the extraction of mandibular third molars using inferior alveolar nerve blocks. Secondary outcomes included, hemodynamic parameters, volume (mL), pain scores assessed during one visit and reported postoperative discomfort assessed during 8 days. Noninferiority was declared if total absence of pain for both groups was equal to 0.9, with a significance level of 5% (P < 0.05) and power at 90% (β = 0.1) at 95% confidence level.

**Results:**

Arteek-SP was applied during the first surgery to 90 volunteers (50.28%) and Septanest was applied to 89 subjects (49.72%). Less than 10% difference was identified, in the proportion of patients with total absence of tooth pain P < 0.05 and in the gum P < 0.1, at 95% CI, when Arteek-SP was applied first in comparison with Septanest, establishing noninferiority.

**Conclusion:**

The clinical performance of the test drug Arteek-SP is noninferior to the reference drug Septanest. They can be considered interchangeable in terms of cost or convenience.

**Registration:**

ClinicalTrials.org, number NCT4166890.

## 1 Introduction

The substitution of drugs is a dilemma present in daily dental procedures, whether it is using original or similar formulations or selecting from different brands [1]. This situation is common in health institutions and practitioners looking for lower cost-benefit drugs but showing apprehension to new ones due to their manufacturing origin and missing scientific information. In order to substitute for a new drug, evidence must be available to check the pharmacokinetic performance of this substitute in relation to the reference drug. Consequently, to decide whether to adopt a new alternative or not, a clinical trial can provide the necessary evidence [2], because patients need effective and safe drugs that are rigorously investigated. The surgical extraction of the third molars using inferior alveolar nerve block (IANB) is one of the most common treatments among dental procedures. There are multiple reasons to perform this procedure, especially because lower third molars are the teeth most frequently included, retained or impacted [3]. Therefore, the use of local anesthetics is essential to perform these treatments. Therefore, pain control during any surgical procedure is one of the most important factors reducing the fear and anxiety associated with a dental procedure [4, 5].

The most common local anesthetic used in dentistry is Articaine, a tertiary amine. Articaine has good liposolubility properties that diffuse easily through soft and hard tissue, exerting adequate pain control [6, 7]. Articaine is regularly used in daily surgeries due to its faster onset, shorter elimination time, rapid recovery from sensory and motor blockade and minimal adverse effects [8]. Additionally, it is assumed to be a safe local anesthetic with few contraindications [1, 9].

The primary aim of this study was to compare non-inferiority of the test formulation Arteek-SP produced by NewStetic, Colombia, on the assumption, it was at least as effective, following IANBs, gum flap, osteotomy, tooth section, extraction and suture, to the reference formulation Septanest produced by Septodont, France. Reported outcomes were on the basis of time, injected volume of solutions and pain. A secondary objective of this study was to evaluate the safety of the test drug compared to the reference drug, regarding hemodynamic parameters, adverse effects, and postoperative complications. Our null hypothesis stated that the sensitivity of pain in patients with the application of the tested drug Arteek is not inferior to the effect of the active control Septanest in the up to 10%.

## 2 Materials and methods

A randomized, triple blind, controlled, crossover clinical trial was carried out on 179 subjects, in compliance with the CONSORT guidelines (Consolidated Standards of Reporting Trials) [10, 11]. Eligibility criteria included both male and female patients between 18 and 25 years of age presenting bilateral asymptomatic mandibular third molars (mesioangular) with the absence of systemic illness. The third molar classification was addressed combining clinical and panoramic x-ray assessment.

Exclusion criteria included medical history suggestive of known or suspected allergies to amides, systemic disease, pregnancy/lactation, subjects who took analgesics 24 h before and presented an episode of infection in the past 6 months.

This study was approved by the School of Dentistry ethics committee (02/2017), University of Antioquia, in accordance with Article 67 of the resolution 008430/93 of the Ministry of Health and Social Protection of the Republic of Colombia and registered in ClinicalTrials.gov (NCT04166890). All volunteers provided written informed consent after attending the study presentation and before any procedure was performed.

The sample size per group (n = 155) was calculated with the two-sample proportion test for Non-Inferiority function (TwoSampleProportion.NIS) of TrialSize Package version 1.4 for R [12]. Based on the results of previous studies [13, 14], the difference in the pain rate of each anesthetic brand (±δ) was specified at ±0.1 and prevalence (p) of success (proportion of patients with total absence of pain) for both groups was equal and defined at 0.9. The level of significance and the power of the trial were adjusted at 5% (P < 0.05) and 90% (β = 0.1), respectively. Therefore, 179 patients were sufficient to declare noninferiority between the brands at 95% confidence level (95% CI).

**Figure 1:**
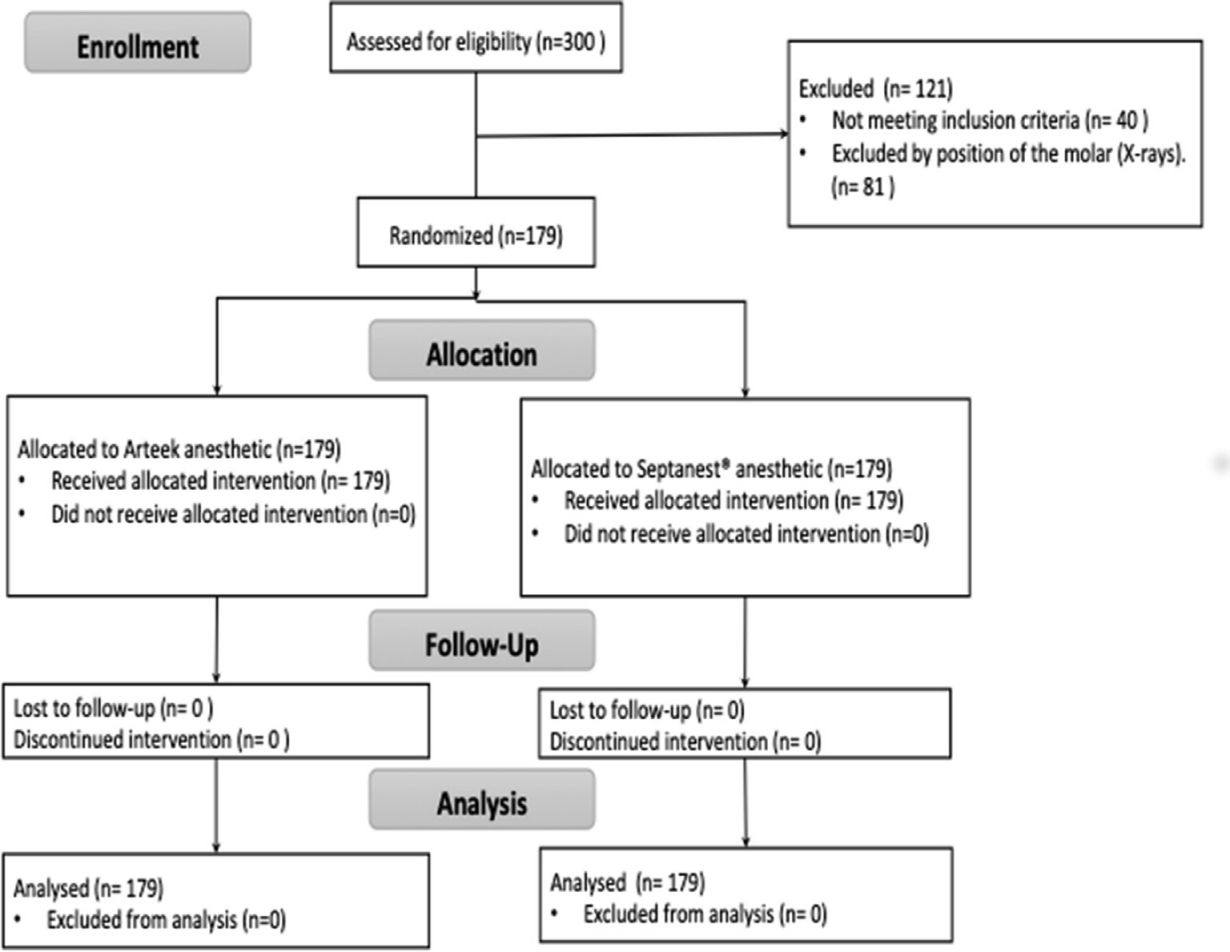
Flow chart of study design and volunteer's recruitment.

The subjects randomly received a combination of IANBs: 4% articaine HCL with 1:100,000 epinephrine (Arteek-SP, NewStetic, Guarne, Colombia) against 4% articaine HCL with 1:100,000 epinephrine (Septanest, Septodont, Saint-Maur-des-Fossés, France) (Figure 1). The study was conducted in full at the Clinics of the School of Dentistry, University of Antioquia, between September 2017 and December 2019, when all the volunteers recruited were attended and the trial was completed successfully.

An independent dentist blinded to the groups, prepared the treatments, color coded the anesthetics and marked them A or B. A pair of cartridges (blue-green) were labeled with the specified side (right or left) and packed in a numbered envelope (1–196); they were then delivered to each patient in order of arrival to the appointment. The treatment groups can be seen in Table 1. It was guaranteed that neither the participants, nor the surgeon, nor the clinical monitor knew about the allocation of treatments at the time of intervention. The team in charge designed the study protocol. Participants were invited to participate and enrolled by the clinical monitor.

**Table 1:** Randomization (Latin square design).

4 Groups 49 unique numbers							
Total 196 participants (392 sides)							
**Group 1** **NS Right Inferior Molar**		**Group 2** **SD Left inferior Molar**		**Group 3** **NS Left inferior Molar**		**Group 4** **SD Right Inferior Molar**	
1	95	4	111	6	117	2	88
5	98	7	118	16	119	3	90
8	99	11	120	18	122	9	94
13	104	12	127	19	125	10	96
21	107	15	134	35	126	14	102
22	108	17	136	36	128	20	103
23	110	26	139	42	132	24	105
29	123	28	140	43	137	25	121
33	130	30	141	44	146	27	124
38	135	31	143	45	148	34	129
41	138	32	150	49	151	37	131
48	147	40	159	59	155	39	133
51	149	47	160	70	157	46	142
54	152	50	161	72	158	52	144
57	156	55	175	81	163	53	145
60	167	58	179	82	165	56	153
73	168	64	183	87	166	61	154
74	169	68	186	89	171	62	162
75	172	71	188	93	174	63	164
76	173	77	190	106	178	65	170
78	176	79	191	112	182	66	181
80	177	92	194	113	187	67	184
84	180	97	195	114	189	69	185
86	196	100		115	192	83	193
91		101		116		85	
		109					

Latin square design was divided into four groups based on 49 unique numbers, total number of volunteers 196 (392 sides), following a Latin square design (18, 23). Group 1 and 3 were allocated with test drug (Articaine Arteek-SP, NewStetic –NS-), Groups 2 and 4 were allocated with the reference test drug (Septanest, Septodont –SP-). Table was designed following a Latin square.

Participants were asked to be present 30 min before the scheduled procedure to record baseline values regarding safety of the solutions. Heart rate (HR), blood pressure (BP) and oxygen saturation (SO2) were recorded before the administration of the anesthetic, 4 min after the injection, when pain was reported and after completion. The participants were instructed to self-report the pain intensity using their hands, when requested during the procedure, following the Heft-Parker visual analog scale (VAS). A scale from 0 to 10 was used, where 0 corresponded to no pain and 10 to unbearable pain [15].

The same surgeon (C–P.L.) performed all the inferior alveolar nerve blocks (IANB). According to the randomization, molar 38 was extracted first followed by molar 48 in the second surgery. Volunteers received 1.8 mL of anesthetic solution (either NewStetic or Septodont). After this injection, every volunteer received an additional injection of 0.7 mL of the same articaine solution into the area to guarantee complete anesthesia of the area. The procedure commenced, when VAS scored 0 for gum, tongue and lip, following a standard protocol [15, 16, 17, 18]. In cases where volunteers reported pain, they were asked to show the value according to the previously explained VAS, and hemodynamic values were immediately measured. Then sufficient complementary anesthesia was injected and the event was recorded. The procedure ended with the suture of the intervened area. Intraoperative bleeding was monitored in all procedures. For postoperative pain management the oral Diclofenac (50 mg every 8 h) was prescribed, during 3 days. The recommended rescue therapy was an injection of Diclofenac (75 mg). To assess clinical postoperative parameters, the clinical monitor scheduled an appointment for suture removal and clinical evaluation of the healing process 7 days after completion of the procedures [19, 20, 21]. Adverse effects were monitored throughout the study period, and all the patients were on follow up for a year.

The primary outcome of the study was to measure the onset of the anesthesia when the tactile and painful sensitivity were blocked (2–6 min after administration of the anesthetics). When the inability to block tactile sensitivity or painful sensitivity after 10 min occurred, or there was a need to use more than two cartridges or reach a dosage of 120 mg of articaine per procedure, it was considered as a failure.

The secondary outcomes were assessed evaluating the dose-response values, injected volume (mL) and time, simultaneously with the visual analog pain scale. Duration of the anesthetic effect was measured 4 min after the initial administration of the anesthetic solutions, until the volunteers expressed sensitivity and pain to stimuli. The safety of Articaine solutions was evaluated by the recording of hemodynamic parameters, and self-reported postoperative discomfort.

Statistical comparisons between the surgery duration, time of latency, onset of the anesthesia, and vital signs were tested non-parametrically using the Kruskal-Wallis test. Differences in the proportions of patients that required complementary anesthesia or reported pain sensation between the anesthetics were evaluated with a two-proportions Z-test. The Wilcoxon test was used to compare the mean VAS score. All statistical tests were carried out at P < 0.05 significance level in R version 1.4 (R Foundation for Statistical Computing, Vienna, Austria).

## 3 Results

A total of 179 healthy volunteers needing bilateral extraction of mandibular third molars with no clinical sign of eruption were included in the study. Ninety-six percent of the participants (both genders) came from low-income socioeconomic backgrounds (Table 2). The summary of measured hemodynamic parameters (heart rate, blood pressure, and saturation of oxygen) can be found in Table 3. Only small differences (P > 0.05) were reported, when comparing the tested drugs before and after completion of the procedure. The study did not reveal statistically significant differences in measured values by either drug along the procedure. However, there was a significant difference when both drugs were compared at certain times between each other. The Septanest showed an increase in heart rate during the surgery compared to Arteek-SP at the level of P < 0.05 (see Table 3). There were no reported adverse effects or harms caused by both tested drugs along the study. At the time of the first scheduled check-up, none of the volunteers requested the use of the rescue therapy. During the suture removal, clinical evaluation of the tissue showed an adequate healing with no signs of inflammation or infection.

**Table 2:** Demographics.

	n	%
Gender		
Male	59	32.96
Female	120	67.04

Age		
18-19	50	27.93
20-21	57	31.84
22-23	40	22.35
24-25	32	17.88
Social/economical strata∗		
1	11	6.15
2	45	25.14
3	117	65.36
4	6	3.35
Anesthetic applied in first surgery		
Arteek	90	50.28
Septanest	89	49.72

∗ The volunteers were classified following local social/economical strata ranging from 1 to 6, (1–2 low income; 3 middle-low; 4 middle income and 5–6 high-income background).

**Table 3:** Vital signs measurements.

	Arteek-SP				Septanest			
	30 min before	Start surgery	During	Completion	30 min before	Start surgery	During	Completion
SBP (mm Hg)	111.08 ± 13.22	112.25 ± 11.43	112.57 ± 11.56	116.89 ± 11.78	112.17 ± 11.63	113.67 ± 11.83	114.73 ± 13.69	118.10 ± 12.90
DBP(mm Hg)	72.88 ± 8.53	71.10 ± 8.16	70.15 ± 8.62	74.94 ± 9.14	74.79 ± 8.16	72.75 ± 8.69	71.57 ± 9.48	75.78 ± 9.34
HR (bpm)	81.51 ± 12.77	83.33 ± 12.83	87.55 ± 13.37	85.77 ± 12.13	81.91 ± 12.04	84.94 ± 13.16	89.91 ± 13.34	86.26 ± 13.13
SO2 (%)	96.35 ± 1.59	96.35 ± 2.14	96.51 ± 2.03	96.69 ± 1.91	96.45 ± 1.59	96.55 ± 1.91	96.83 ± 1.62	96.74 ± 1.57

SBP: systolic blood pressure, DBP: diastolic blood pressure, HR: heart rate. Significant differences according to Kruskal-Wallis test between anesthetics in each measure at P < 0.05 (∗).

**Figure 2:**
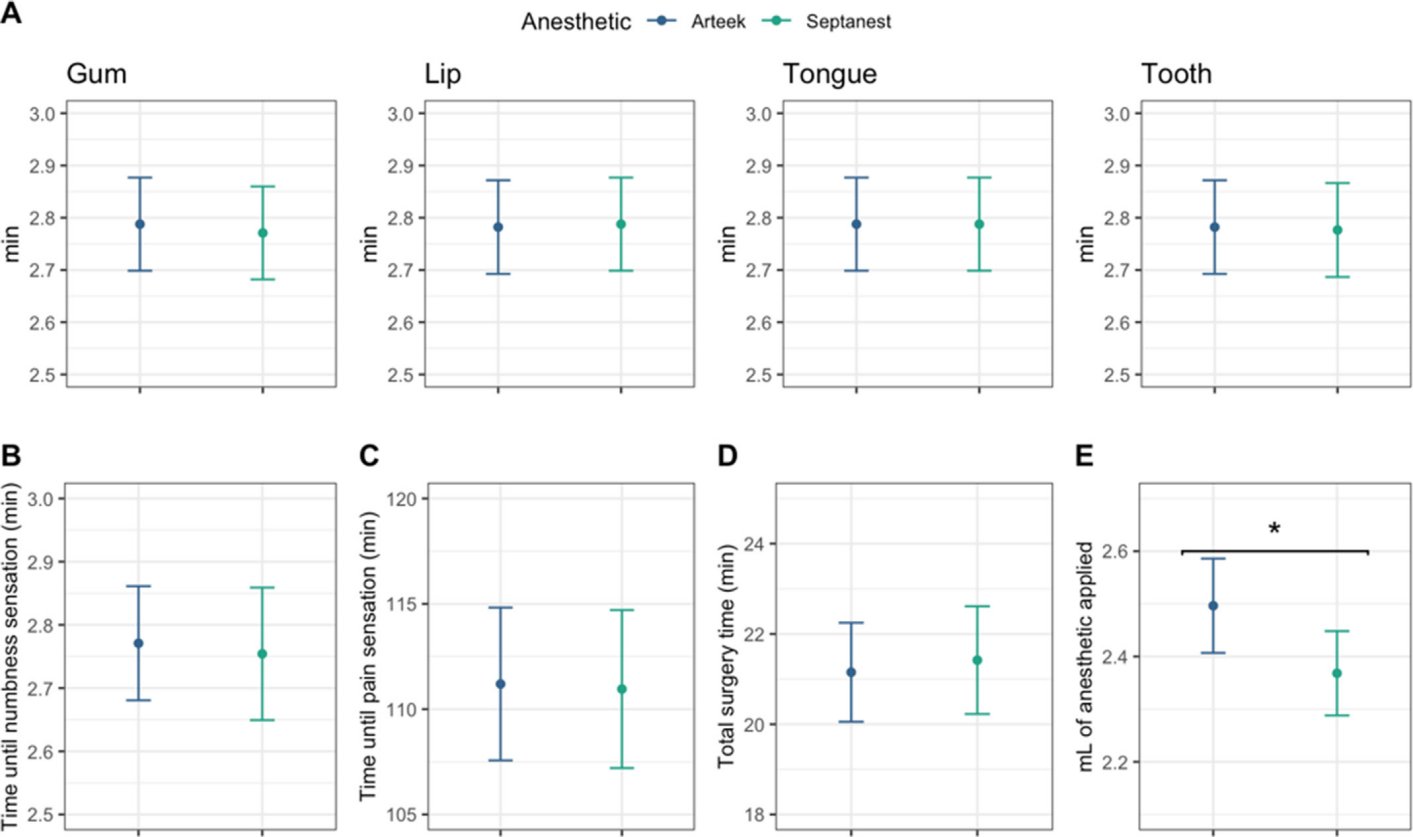
Comparison of Arteek-SP and Septanest solutions with 95% CI. A. Time of latency in the different mouth zones. B. Onset of the anesthesia (primary outcome). C. Time until pain sensation appeared (secondary outcome). D. Total surgery duration. E. Volume of applied anesthetic. Comparisons marked with an asterisk are significantly different (P < 0.05) based on a Kruskal-Wallis test.

The duration (latency) of both anesthetics showed very similar and non-significant results (P > 0.05) in different mouth zones – gum, lip, tongue, and tooth as depicted in Figure 2A. Moreover, no significant differences were observed neither in onset times between the brands of anesthetics measuring the time until numbness sensation appeared (Arteek-SP 2.77 ± 0.09 min; Septanest 2.75 ± 0.10min) (Figure 2B), nor the measured time between the anesthetics' application and pain sensation (residual analgesia), where the measured average time was 111.19 ± 3.62 min for Arteek-SP and 110.95 ± 3.75 min for Septanest, respectively (see Figure 2C). Also, the total surgery duration did not reveal any significant difference between the two anesthetics (21.15 ± 0.56 min for Arteek-SP and 21.42 ± 0.61 min for Septanest) (Figure 2D).

**Figure 3:**
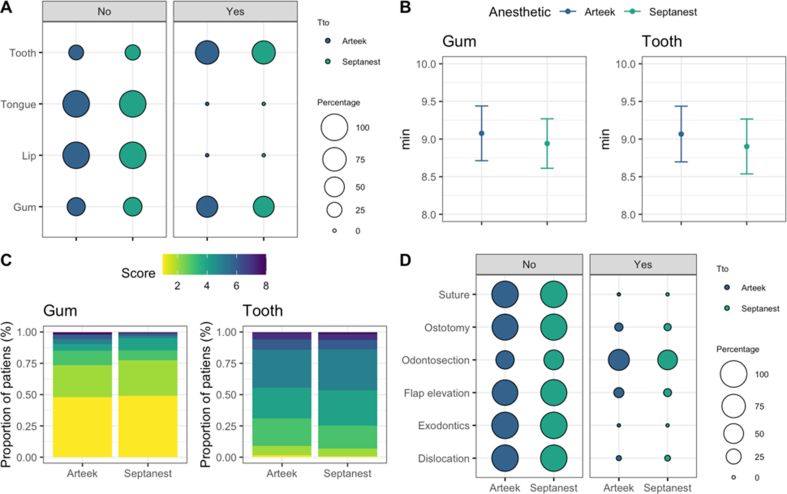
Comparison of pain scores of Arteek-SP and Septanest solutions. A. Proportion of patients with and without pain sensation in different mouth zones. B. Minutes between anesthetic application and pain sensation with 95% confidence intervals C. VAS pain scale. D. Proportion of patients that require complementary anesthesia at different steps of the surgery.

However, the average of injected volume of anesthetic revealed a significant difference between Arteek-SP and Septanest (0.131 mL) (Figure 2E), where the mean volume of Arteek-SP was 2.49 ± 0.09 mL while for Septanest was 2.38 ± 0.08 mL (P < 0.05). This result corresponds to the fact that during the different stages of surgery (i.e., flap elevation, osteotomy, tooth section) the proportion of patients who required complementary anesthesia was higher for Arteek-SP anesthesia, although the differences were not statistically significant. The surgery step in which patients required complementary anesthesia more frequently was the tooth section, where 58.10% (Arteek-SP) and 49.72% (Septanest) subjects received complementary anesthetic injection (Figure 3D). Intraoperative bleeding remained minimal during all surgeries. It is important to note that although the difference in volume (0.131 mL) was statistically significant, it was not considered clinically relevant.

During tooth section, 74% of volunteers reported pain and 58% of them in the gum at 9.08 ± 0.19 min for Arteek-SP and 8.94 ± 0.17 min for Septanest (Figure 3A, 3B). Average VAS score for pain for the gum was 1.25 (Arteek-SP) and 1.12 (Septanest), while for the tooth was 3.94 (Arteek-SP) and 3.91 (Septanest) (Figure 3C). All of these results were found non-significant comparing Arteek-SP to Septanest.

## 4 Discussion

Most of the participants of this study were considered as vulnerable students needing extra support with a higher number of females participating in the study (Table 1), however no significant differences were observed regarding gender. All procedures were performed in one visit, minimizing the financial burden on the volunteers, which were then provided access to prime drugs not regularly used by the public health service, due to elevated cost or simply by unawareness and these were free of charge.

In the present study, the hemodynamic parameters were statistically insignificant before and after the completion of extraction (P > 0.05). There was a heart rate increase during the first 10 min caused by the application of both brands and gradually decreased to the initial values after the completion of the procedure. These results were congruent to previous studies reported by other authors [22, 23, 24] An increase in heart rate after injection was likely caused by increased endogenous catecholamine because of the pain induced from the injection, as shown by Meral et al. [25]. It is worthy to note that the study did not evaluate the nutritional conditions, which could affect the interindividual responses in the measurements of hemodynamic parameters.

As previously reported by other authors, there was a sudden hypotension recorded soon after the administration of the Articaine solutions (Arteek-SP and Septanest). It returned to baseline values within an hour after the injection. There were no statistical differences in the values of the systolic and diastolic blood pressure 1 h after (P > 0.05) [24, 25]. The similarity of physiological responses (hemodynamic parameters) in both treated groups showed a normal biological response to articaine and to the clinical environment, supporting the evidence of the safety of Articaine solutions.

Pain measurement is always difficult to establish, because its perception and intensity are multifactorial, however the direct self-report is the most common in clinical practices [7]. The clinical settings provided a minimum comfort for patient and surgeon; also factors such as noise, temperature and a busy environment were always a concern, making it difficult to exclude the influence of stress on volunteers. These conditions could possibly increase acute stressors, such as cortisol and catecholamine as reported by Russell & Lightman [26].

The onset of the anesthesia and pain control are consistent with other reports evaluating 4% Articaine [27, 28]. The minimum possible volume was used to block the inferior alveolar nerve and buccal nerve. Nonetheless, it was necessary to use more volume of Arteek-SP compared to Septanest (Figure 2E). Although it was clinically imperceptible, the difference was statistically significant (P < 0.04). The presence of sodium metabisulfite included as an antioxidant in commercial formulas of local anesthetics [29] extends the half-life of epinephrine in the body [8, 30]. Thus, it might play a role modifying the volume of solution injected. It is speculated that different concentrations of preservatives in the formulas might be the reason for the differences in the volumes used, prolonging the anesthesia when Septanest was the first anesthetic injected and the opposite for Arteek-SP. Consequently, the volumetric difference was in favor of the reference drug Septanest. This outcome could affect the duration of the anesthetic effect, proving superiority of the reference drug (Septanest) in a secondary endpoint (anesthesia duration). However, the total surgery time and the time until pain sensation after injection did not reveal any significant differences (Figure 2C, D). One highly experienced surgeon performed all the interventions, which positively facilitated standardized procedures and the precise administration of the anesthetics and reduced multiple operator bias. This could also make small technical adaptations masking possible pharmacokinetic variations between the compared brands (Figure 3A, B, D); however, this specific bias could not be addressed. Another possibility for the volumetric difference could be the materials and fabricating processes each manufacturer employed (stiffer rubber plunger, rubber diaphragm or quality of the glass tube). Nevertheless, there was no need to use more than two cartridges of anesthesia per procedure; therefore no data were excluded from the study.

Furthermore, the use of the visual analog scale, which is a widely used and proven method, still presents challenges to measure pain, due to its individual perception and multifactor causes (Figure 3C). Thus, further trials should include sampling saliva or blood to analyze pain related molecular markers along with the analog scale of Heft-Parker to reduce possible bias.

The proportion of patients with total absence of pain, showed less than 10% difference. In conclusion, the results of this study showed that the test drug Arteek-SP was within the margin calculated compared to the reference drug Septanest establishing noninferiority. This study provides valuable information to make an evidence-based decision to adopt or substitute anesthetic brands in daily clinical practice for effective management of surgical tooth extraction, in terms of availability, cost or convenience.

## Declarations

### Author contribution statement

David Arboleda-Toro: Conceived and designed the experiments; Wrote the paper.

Liliana Castrillon-Pino: Performed the experiments.

Yuliana Andrea Osorio Osorno: Contributed reagents, materials, analysis tools or data.

Lenka Toro: Analyzed and interpreted the data; Wrote the paper.

Nathalia Maria Vanessa Florez-Zapata: Analyzed and interpreted the data.

### Funding statement

This work was supported by New Stetic S.A., Guarne, Colombia.

### Data availability statement

Data will be made available on request.

### Declaration of interests statement

The authors declare no conflict of interest.

### Additional information

The clinical trial described in this paper was registered at ClinicalTrials.gov under the registration number NCT04166890.

No additional information is available for this paper.

## Appendix A Supplementary data

CONSORT_2010_Checklist CONSORT_2010_Checklist

## References

[1] St George G., Morgan A., Meechan J., Moles D.R., Needleman I., Ng Y.-L., Petrie A. (2018). Injectable local anaesthetic agents for dental anaesthesia. Cochrane Database Syst. Rev..

[2] Head S.J., Kaul S., Bogers A.J.J.C., Kappetein A.P. (2012). Non-inferiority study design: lessons to be learned from cardiovascular trials. Eur. Heart J..

[3] Patel S., Mansuri S., Shaikh F., Shah T. (2017). Impacted mandibular third molars: a retrospective study of 1198 cases to assess indications for surgical removal, and correlation with age, sex and type of impaction-A single institutional experience. J. Maxillofac. Oral Surg..

[4] Appukuttan D.P. (2016). Strategies to manage patients with dental anxiety and dental phobia: literature review. Clin. Cosmet. Invest. Dent..

[5] Araújo R.Z., Pinto Júnior A.A.C., Sigua-Rodriguez E.A., Olate S., Fonseca Alves L.C., de Castro W.H. (2016). Pain control in third molar surgery. Int. J. Odontostomatol.

[6] Becker D.E., Reed K.L. (2006). Essentials of local anesthetic pharmacology. Anesth. Prog..

[7] da Silva-Junior G.P., de Almeida Souza L.M., Groppo F.C. (2017). Comparison of articaine and lidocaine for buccal infiltration after inferior alveolar nerve block for intraoperative pain control during impacted mandibular third molar surgery. Anesth. Prog..

[8] Bajwa S.J.S., Jindal R. (2012). Use of Articaine in loco-regional anesthesia for day care surgical procedures. J. Anaesthesiol. Clin. Pharmacol..

[9] Snoeck M. (2012). Articaine: a review of its use for local and regional anesthesia. Local Reg. Anesth..

[10] Piaggio G., Elbourne D.R., Pocock S.J., Evans S.J.W., Altman D.G. (2012). Reporting of noninferiority and equivalence randomized trials: extension of the CONSORT 2010 statement. J. Am. Med. Assoc..

[11] Schulz K.F., Altman D.G., Moher D. (2010). CONSORT 2010 statement: updated guidelines for reporting parallel group randomised trials. J. Pharmacol. Pharmacother..

[12] Zhang Q.E. (2014). R functions in Chapter 3,4,6,7,9,10,11,12,14,15. Packag. ‘TrialSize,’.

[13] Hintze A., Paessler L. (2006). Comparative investigations on the efficacy of articaine 4% (epinephrine 1:200,000) and articaine 2% (epinephrine 1:200,000) in local infiltration anaesthesia in dentistry--a randomised double-blind study. Clin. Oral Invest..

[14] Yapp K.E., Hopcraft M.S., Parashos P. (2011). Articaine: a review of the literature. Br. Dent. J..

[15] Cook O., Nusstein J., Drum M., Fowler S., Reader A., Draper J. (2018). Anesthetic efficacy of a combination of 4% prilocaine/2% lidocaine with epinephrine for the inferior alveolar nerve block: a prospective, randomized, double-blind study. J. Endod..

[16] Engelke W., Beltrán V., Cantín M., Choi E.-J., Navarro P., Fuentes R. (2014). Removal of impacted mandibular third molars using an inward fragmentation technique (IFT) - method and first results. J. Cranio-Maxillo-Facial Surg. Off. Publ. Eur. Assoc. Cranio-Maxillo-Facial Surg..

[17] Senes A.M., Calvo A.M., Colombini-Ishikiriama B.L., Gonçalves P.Z., Dionísio T.J., Sant’ana E., Brozoski D.T., Lauris J.R.P., Faria F.A.C., Santos C.F. (2015). Efficacy and safety of 2% and 4% articaine for lower third molar surgery. J. Dent. Res..

[18] Ali A.S., Benton J.A., Yates J.M. (2018). Risk of inferior alveolar nerve injury with coronectomy vs surgical extraction of mandibular third molars-A comparison of two techniques and review of the literature. J. Oral Rehabil..

[19] Colombini B.L., Modena K.C.S., Calvo A.M., Sakai V.T., Giglio F.P.M., Dionísio T.J., Trindade A.S.J., Lauris J.R.P., Santos C.F. (2006). Articaine and mepivacaine efficacy in postoperative analgesia for lower third molar removal: a double-blind, randomized, crossover study. Oral Surg. Oral Med. Oral Pathol. Oral Radiol. Endod..

[20] Santos C.F., Modena K.C.S., Giglio F.P.M., Sakai V.T., Calvo A.M., Colombini B.L., Sipert C.R., Dionísio T.J., Faria F.A.C., Trindade A.S.J., Lauris J.R.P. (2007). Epinephrine concentration (1:100,000 or 1:200,000) does not affect the clinical efficacy of 4% articaine for lower third molar removal: a double-blind, randomized, crossover study. J. Oral Maxillofac. Surg. Off. J. Am. Assoc. Oral Maxillofac. Surg..

[21] Gregorio L.V.L., Giglio F.P.M., Sakai V.T., Modena K.C.S., Colombini B.L., Calvo A.M., Sipert C.R., Dionísio T.J., Lauris J.R.P., Faria F.A.C., Trindade Junior A.S., Santos C.F. (2008). A comparison of the clinical anesthetic efficacy of 4% articaine and 0.5% bupivacaine (both with 1:200,000 epinephrine) for lower third molar removal., Oral Surg. Oral Med. Oral Pathol. Oral Radiol. Endod..

[22] Moore P.A., Boynes S.G., V Hersh E., DeRossi S.S., Sollecito T.P., Goodson J.M., Leonel J.S., Floros C., Peterson C., Hutcheson M. (2006). The anesthetic efficacy of 4 percent articaine 1:200,000 epinephrine: two controlled clinical trials. J. Am. Dent. Assoc..

[23] de H Vasconcellos R.J., Vasconcelos B.C. do E., Genú P.R. (2008). Influence of local anesthethics with adrenalina 1:100.000 in basic vital constants during third molar surgery. Med. Oral Patol. Oral Cir. Bucal.

[24] Kämmerer P.W., Palarie V., Daubländer M., Bicer C., Shabazfar N., Brüllmann D., Al-Nawas B. (2012). Comparison of 4% articaine with epinephrine (1:100,000) and without epinephrine in inferior alveolar block for tooth extraction: double-blind randomized clinical trial of anesthetic efficacy. Oral Surg. Oral Med. Oral Pathol. Oral Radiol..

[25] Meral G., Tasar F., Sayin F., Saysel M., Kir S., Karabulut E. (2005). Effects of lidocaine with and without epinephrine on plasma epinephrine and lidocaine concentrations and hemodynamic values during third molar surgery. Oral Surg. Oral Med. Oral Pathol. Oral Radiol. Endod..

[26] Russell G., Lightman S. (2019). The human stress response. Nat. Rev. Endocrinol..

[27] Shapiro M.R., McDonald N.J., Gardner R.J., Peters M.C., Botero T.M. (2018). Efficacy of articaine versus lidocaine in supplemental infiltration for mandibular first versus second molars with irreversible pulpitis: a prospective, randomized, double-blind clinical trial. J. Endod..

[28] Silva S.A., Horliana A.C.R.T., Pannuti C.M., Braz-Silva P.H., Bispo C.G.C., Buscariolo I.A., Rocha R.G., Tortamano I.P. (2019). Comparative evaluation of anesthetic efficacy of 1.8 mL and 3.6 mL of articaine in irreversible pulpitis of the mandibular molar: a randomized clinical trial. PloS One.

[29] Grubstein B., Milano E. (1992). Stabilization of epinephrine in a local anesthetic injectable solution using reduced levels of sodium metabisulfite and edta. Drug Dev. Ind. Pharm..

[30] Haas D.A. (2002). An update on local anesthetics in dentistry. J. Can. Dent. Assoc..

